# Trial Forge Guidance 1: what is a Study Within A Trial (SWAT)?

**DOI:** 10.1186/s13063-018-2535-5

**Published:** 2018-02-23

**Authors:** Shaun Treweek, Simon Bevan, Peter Bower, Marion Campbell, Jacquie Christie, Mike Clarke, Clive Collett, Seonaidh Cotton, Declan Devane, Adel El Feky, Ella Flemyng, Sandra Galvin, Heidi Gardner, Katie Gillies, Jan Jansen, Roberta Littleford, Adwoa Parker, Craig Ramsay, Lynne Restrup, Frank Sullivan, David Torgerson, Liz Tremain, Matthew Westmore, Paula R. Williamson

**Affiliations:** 1 Health Services Research Unit, University of Aberdeen, Aberdeen, UK; 2 National Institute for Health Research Evaluation, Trials and Studies Coordinating Centre, University of Southampton, Southampton, UK; 3 Centre for Primary Care, University of Manchester, Manchester, UK; 4 GSK Medicines Research Centre, Stevenage, UK; 5 Northern Ireland Network for Trials Methodology Research, Queen’s University Belfast, Belfast, UK; 6 Health Research Authority, London, UK; 7 HRB-Trials Methodology Research Network, National University of Ireland Galway, Galway, Ireland; 8 BioMed Central, London, UK; 9 Division of Acute Care Surgery, University of Alabama at Birmingham, Birmingham, AL USA; 10 Tayside Clinical Trials Unit, University of Dundee, Dundee, UK; 11 York Trials Unit, University of York, York, UK; 12 Aberdeen, UK; 13 School of Medicine, St Andrews University, St Andrews, UK; 14 MRC North West Hub for Trials Methodology Research, Department of Biostatistics, University of Liverpool, Liverpool, UK

## Abstract

Randomised trials are a central component of all evidence-informed health care systems and the evidence coming from them helps to support health care users, health professionals and others to make more informed decisions about treatment. The evidence available to trialists to support decisions on design, conduct and reporting of randomised trials is, however, sparse. Trial Forge is an initiative that aims to increase the evidence base for trial decision-making and in doing so, to improve trial efficiency.

One way to fill gaps in evidence is to run Studies Within A Trial, or SWATs. This guidance document provides a brief definition of SWATs, an explanation of why they are important and some practical ‘top tips’ that come from existing experience of doing SWATs. We hope the guidance will be useful to trialists, methodologists, funders, approvals agencies and others in making clear what a SWAT is, as well as what is involved in doing one.

## Introduction

Randomised trials are a central component of all evidence-informed health care systems and they form a body of evidence that can help health care users, health professionals, policy-makers and others make informed choices about the effectiveness of treatments and therapies that they use and provide. The same is not true for trial design, conduct and reporting decisions, which are generally uninformed by evidence because there is little relevant evidence to turn to.

Trial Forge (www.trialforge.org) [1] is an initiative that aims to increase the evidence base for trial decision-making and, in doing so, to improve trial efficiency. One way to fill gaps in evidence is to run Studies Within A Trial, or SWATs. Descriptions of SWATs have been published [2, 3] but here we provide some guidance that provides a brief definition of a SWAT, an explanation of why they are important and some practical ‘top tips’ that come from existing experience of doing SWATs. We hope the guidance will be useful to trialists, methodologists, funders, approvals agencies and others in making clear what a SWAT is, as well as what is involved in doing one. We encourage them to use the text freely on their own websites and materials, with appropriate acknowledgement.

The text is based on discussions held during and after a 1-day meeting in Aberdeen, UK on 23 March 2017 as part of the Trial Forge initiative. This paper is the first Trial Forge Guidance document and there will be more guidance documents in the future, each providing what we hope is clear help and guidance around an issue relevant to improving the evidence base for trial decision-making. Trial methodologists and other stakeholders will be consulted to determine the topic areas and scope for future guidance.

## What is a Study Within A Trial (SWAT)?

A SWAT is a self-contained research study that has been embedded within a host trial with the aim of evaluating or exploring alternative ways of delivering or organising a particular trial process.

## Why do we need to do SWATs?

The need for randomised trials to evaluate the effects of health care interventions, such as new drugs and other treatments, is a familiar concept to people working in health and health research. The result of the trial provides evidence on how effective (or ineffective) the intervention is, helping both practitioners and health care users to make well-informed decisions about using it. These trials are central to improvements in health and social care.

Therefore, it is essential that the trials themselves are done in the most effective ways and one way to do this is to use the same types of evaluation to investigate and improve the processes of how we do randomised trials.

Unfortunately, only a small number of such studies have been done and there is very little evidence to allow researchers to make well-informed decisions about how to do their trials [1]. This means that researchers doing trials, funders paying for them and patients taking part in them cannot always be sure that the way the trial is being done is as effective and efficient as it could be. The most obvious example of this is that the evidence available to support trial teams to recruit patients to their trials is very thin, despite recruitment being a recognised problem for many trials [4, 5] and being identified as the top priority for research into trial methods [6].

One way of increasing this evidence base is to do a Study Within A Trial (SWAT) [2, 3]. SWATs evaluate alternative ways of doing a trial process (e.g. recruiting patients, helping them to stay in the study, or reporting the findings) to provide evidence about how to improve the process.

### Key features of a typical SWAT

- It seeks to resolve important uncertainties about the processes used in trials
- It is embedded within a host trial
- It must not affect the scientific integrity of the host trial, its rationale or outcome measures
- It should have a formal protocol, just like the host trial
- It can be evaluated in a single trial but is well-suited for running across more than one host trial, either at the same time or sequentially
- It will provide data to inform the design and conduct of future trials but might also provide data to inform decisions about the ongoing host trial

For some practical considerations regarding SWATs, see Table 1. The information in the table comes mainly from experience with SWATs in the UK and Ireland but is likely to be useful for SWATs planned in other countries too.

**Table 1:** Practical things to consider when planning a Study Within A Trial (SWAT)

Cost
• SWATs need not be expensive; our experience is that many are likely to cost between £5000 and £10,000. Ideally, they should be built into the host trial from the start and the associated costs can be included in the budget for the host trial. If the findings of the SWAT will be reported in a standalone publication in an author-pays open-access journal, the costs of this will be need to be budgeted for
Randomisation
• Whether randomisation is needed depends on the question being asked. If the intention is to evaluate the effect of alternative ways of doing a trial process, then the alternatives being compared should be allocated at random. This may not always be possible and another allocation method (e.g. before and after the new alternative) can be used but in most cases this will weaken confidence in the results. However, if the question being asked is not focused on measuring effect sizes (e.g. it could be concerned with understanding why something is done the way it is) then randomisation is likely to be inappropriate and other qualitative methods would be required. Randomisation is not a defining feature of a SWAT
• Randomisation can be by a separate process to that used for the host trial randomisation
Ethics
• Ethical approval guidelines and regulations for conducting research in humans vary between countries. Depending on the specific SWAT protocol being evaluated, it is advised that the researcher checks national guidance and discusses whether ethical approval is required with their institutional or local ethical committee
• It is likely that some, but not all, SWATs will need ethical and other approvals. Clinical trials of medicinal products in the EU are provided for in Directive 2001/20/EC of the European Parliament. Such trials require research ethical approval and it is likely that any SWAT within a host trial subject to the EU directive will require ethical review
• Ethical governance of clinical trials outside of the directive, i.e. non-medicinal products for human use vary between countries. In the UK for instance, SWATs within non-medicinal product trials that involve only trial staff will not normally need UK NHS ethical approval (but may need institutional review), while it is likely that those that involve NHS patients will. In the Republic of Ireland, there is a system of national approval for trials of medicinal products but not for non-medicinal products and, therefore, for the latter ethical approval is usually sought from sites conducting the host trial and/or from the SWAT principal investigator’s host institution. If the SWAT was planned at the same time as the host trial, then it could be included in the application for ethical approval of the host trial. Trial Forge has a collection of material that has been used before to obtain ethical approval for a SWAT, which it adds to its own SWAT packages. Contact info@trialforge.org for more details
• SWATs are generally low risk and it is rare for them to impose additional burden or risk on participants and consequently it will not usually be necessary to get individual consent from participants. Indeed, in many cases individual consent may not be appropriate. It may confuse patients as to what they are consenting to, and may impact on their behaviour if they are aware that different recruitment methods are being tested, confounding the evaluation
• SWATs aimed at staff, but which directly affect patients/participants, may need NHS or other ethical review (e.g. studies that change what recruiters say to potential participants, or who says it to them). Where there is any doubt researchers should contact the Health Research Authority (HRA)/Devolved Nation’s REC administrative body to check whether NHS ethical review is required. In the Republic of Ireland, researchers should check with the ethics committee approving the host trial and with any site in which the trial will be conducted
• In the UK NHS Research Ethics Committee approval is not normally required for research only involving staff who are recruited by virtue of their professional role. However, where such studies are led from England and involves the NHS in England ‘HRA approval’ may be required (see http://www.hra.nhs.uk/about-the-hra/our-plans-and-projects/assessment-approval/ for further information)
Analysis
• The analysis of SWATs might be simple (such as the comparison of two proportions) and might be done by members of the trial team other than a senior statistician
• Sample size calculations for SWATs can be done in the usual way using estimates of minimum important differences that the investigators or others consider appropriate. The size of a SWAT is constrained by the host trial. The size of a recruitment SWAT will generally be larger than the host trial sample size (the constraint is the size of the patient population approached, not recruited). Other SWATs (such as those on retention) will be limited to the actual host trial sample. It is highly unlikely that the size of the host trial will be changed for the benefit of a SWAT. SWATs are designed for future meta-analysis. In other words, while an individual SWAT may be underpowered, a meta-analysis of several well-done SWATs evaluating the same intervention and following the same protocol can provide compelling evidence for trial process decision making. As with all meta-analysis, judgements need to be made about whether it is sensible to combine studies done in different populations, disease areas and settings. This issue will be the topic of future Trial Forge Guidance
• SWATs exploring qualitative questions about how a trial process is delivered, organised or perceived will be analysed using a suitable qualitative analysis method
Implementing the SWAT
• Some of the extra work needed for a SWAT (e.g. putting additional materials or incentives into envelopes along with information leaflets) might be done by temporary staff, or existing staff who have a lull in the work for their own trial. For other SWATs there might be little additional work involved (for example, using mail merge software to generate different invitation letters). However, confidentiality/data protection issues may limit who can do the work, depending on its content and the potential for identifying participants to those who would not otherwise have lawful access to personal identifiable information
• They need not run for the whole duration of the host trial so any extra work may be both modest and short-term
Publication
• The findings of the SWAT should be put into the public domain and should be accessible by others. This might be possible through inclusion in the report of the host trial (with appropriate signposting, perhaps in the abstract, to highlight its presence), in a standalone dedicated publication or through inclusion in a relevant systematic review

### An example of a SWAT

Most trials have a Participant Information Leaflet (PIL), which tells a potential participant about the trial. The trial team uses this to offer information to potential trial participants in a way that it hopes will also help recruitment (and perhaps retention) whilet adhering to ethical standards.

**Fig. 1:**
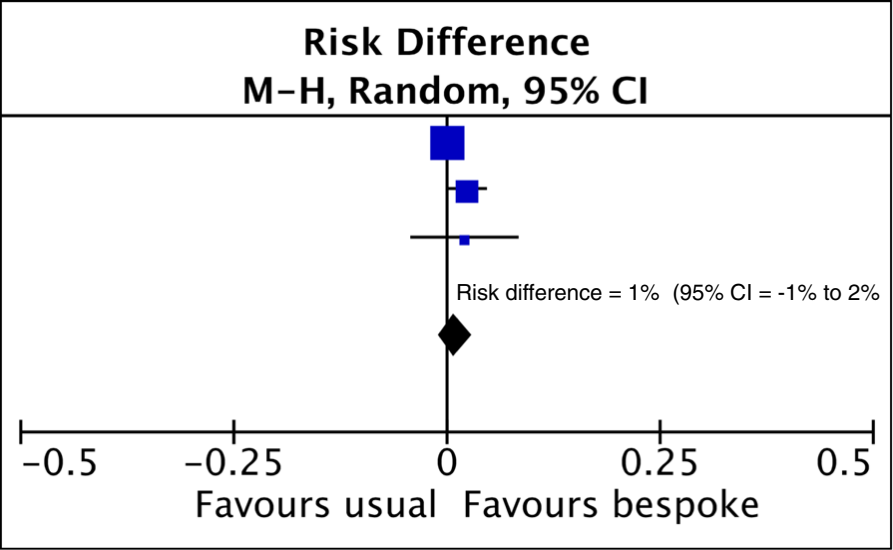
Meta-analysis of three evaluations of the effect on trial recruitment of a bespoke, tailored and user-tested method of developing a Participant Information Leaflet

The Systematic Techniques for Assisting Recruitment to Trials (START) programme (http://research.bmh.manchester.ac.uk/mrcstart/) developed a SWAT to evaluate the effect of a bespoke, tailored and user-tested PIL on recruitment compared with a standard PIL. The bespoke method of developing a PIL is expensive so it is important to know how much, if any, difference it makes to the trial. For instance, if the aim is to increase recruitment, it is essential to know that recruitment is indeed increased with the bespoke PIL compared with a standard PIL before using it in a future trial. The SWAT has already been evaluated in several trials (see, for example [7, 8]) and the emerging results are shown in Fig. 1. This meta-analysis shows that the current estimate for the effect on recruitment is small and not statistically significant: 1% improvement (95% confidence interval, − 1–2%).

In other words, the bespoke PIL had little or no effect on recruitment compared with a standard PIL. By approaching investigators, encouraging them to embed an evaluation of the two types of PIL into their trials and then coordinating the analysis of data from those trials that did, the START programme’s coordinated, collaborative approach of embedding a SWAT evaluation in trials involving over 6600 people now provides an evidence base for researchers trying to decide on whether to develop a bespoke PIL for their trial.

Other examples of questions that could be addressed in SWATs include:

- Comparing the effect of different financial incentives to encourage patients to complete a questionnaire used to collect trial outcomes
- Determining whether recruitment is boosted if non-responders to postal invitations to join a trial are reminded by telephone
- Evaluating the effect on recruitment and retention of a two-stage Participant Information Leaflet (i.e. the leaflet is delivered to participants in two parts: a short ‘key points’ version together with a longer version containing more detail) compared with a standard, single-stage leaflet
- Evaluating the effect on data quality of providing site staff with face-to-face data entry training compared with Skype or video-conference training
- Exploring which type of information participants think would best recognise the value of their contribution to the host trial results

There are plenty of uncertainties around how we should do trials, so it is highly likely that a trial team can find something that is interesting to them and worth investigating in a SWAT. For example, the Prioritising Recruitment in Randomised Trials (PRioRiTy) project (http://priorityresearch.ie/) generated a list of priority areas for recruitment research and many of these could be addressed by SWATs.

### What happens to SWAT results?

Just as health researchers have a responsibility to make the findings of their clinical trials available, the findings from SWATs should be made public, so that the evidence base available for future decisions can increase. The findings can then be picked up by systematic reviewers and others who synthesise research evidence. The person doing the SWAT can facilitate this by, for example, contacting those who have done a relevant Cochrane Methodology Review (who will be updating it), to let them know about the SWAT. This means that even if the SWAT is not published separately itself, its results can be incorporated into the review.

SWAT results can also directly inform decisions within the host trial where uncertainty exists as to the best method to use for a particular process. The BEEP trial (https://www.journalslibrary.nihr.ac.uk/programmes/hta/126712/#/) is using a SWAT [9] and an interim analysis to help make a decision about the retention strategies to be used in the trial. A web-based trial linked to antibiotic prescribing also used a SWAT to make a decision about the best way to invite participants to take part in the second stage of the trial [10]. Although both of these SWATs provide useful information for other trials, they were designed to directly inform process decisions taken within the host trial.

### The SWAT repository

Queen’s University Belfast in Northern Ireland hosts a SWAT repository (go.qub.ac.uk/SWAT-SWAR), which contains a list of prepared SWAT outlines. A form to register a new SWAT is also available online at http://www.qub.ac.uk/sites/TheNorthernIrelandNetworkforTrialsMethodologyResearch/SWATSWARInformation/ApplicationForms/SWATApplication/. Registering SWATs on the repository helps to avoid unnecessary duplication of effort and provides other researchers with ideas for how they might test the processes they will use in their own clinical trial.

### The PRioRiTy repository

If your SWAT addresses one of the top 20 PRioRiTy research questions it can also be added to the PRioRiTy online repository (http://priorityresearch.ie/) which is a collection of ongoing research specific to recruitment to trials. This repository is hosted by the Health Research Board – Trials Methodology Research Network in Ireland. Ideally, these methodology studies should be included in both the PRioRiTy and the SWAT repository to help people to find them.

## Abbreviations

PIL: Participant Information Leaflet
PRioRiTy: Prioritising Recruitment in Randomised Trials
START: Systematic Techniques for Assisting Recruitment to Trials
SWAT: Study Within A Trial
